# Host-Specific Activation of Entomopathogenic Nematode Infective Juveniles

**DOI:** 10.3390/insects9020059

**Published:** 2018-06-02

**Authors:** Valentina Alonso, Shyon Nasrolahi, Adler R. Dillman

**Affiliations:** Department of Nematology, University of California, Riverside, CA 92521, USA; valon001@ucr.edu (V.A.); snasr001@ucr.edu (S.N.)

**Keywords:** Entomopathogenic nematode, activation, Steinernema, infective juveniles

## Abstract

Entomopathogenic nematodes (EPNs) are potent insect parasites and have been used for pest control in agriculture. Despite the complexity of the EPN infection process, hosts are typically killed within 5 days of initial infection. When free-living infective juveniles (IJs) infect a host, they release their bacterial symbiont, secrete toxic products, and undergo notable morphological changes. Collectively, this process is referred to as “activation” and represents the point in a nematode’s life cycle when it becomes actively parasitic. The effect of different host tissues and IJ age on activation, and how activation itself is related to virulence, are not well understood. Here, we employed a recently developed bioassay, which quantifies IJ activation, as a tool to address these matters. Appreciating that activation is a key part of the EPN infection process, we hypothesized that activation would positively correlate to virulence. Using the EPNs *Steinernema carpocapsae* and *S. feltiae* we found that EPN activation is host-specific and influenced by infective juvenile age. Additionally, our data suggest that activation has a context-dependent influence on virulence and could be predictive of virulence in some cases such as when IJ activation is especially low.

## 1. Introduction

Entomopathogenic nematodes (EPNs) are a guild of insect-parasitic nematodes that kill their hosts rapidly and utilize pathogenic bacteria to facilitate their parasitic lifestyle [1,2]. EPNs have applied uses such as the biological control of pests [3,4], but they are also useful models for studying a wide variety of biology including bacterial-host symbiosis [5,6], and as potential models for vertebrate parasites [7]. The infection process for EPNs is complex and includes several steps: Infective juveniles (IJs) must first find a host using long-term and short-term cues, penetrate the host, and activate within the host to release their toxins and symbiotic bacteria, all before finally killing the host [1,8,9,10]. Activation results in physiological changes and initiates an EPN’s actively parasitic lifestyle [7,11]. In a recent study, in vitro activation experiments were used to further support the long-standing notion that the EPN *Steinernema scapterisci* is a cricket specialist [11]. Here we used the same technique to understand the activation dynamics of *S. carpocapsae* and *S. feltiae* IJs when exposed to the tissue of a variety of potential insect hosts. *Steinernema carpocapsae* and *S. feltiae* are two of the best studied members of the genus *Steinernema*—which contains more than 80 described species, many of which are considered generalists [12,13,14]. We then assessed the predictive value of IJ activation by conducting virulence assays on sand crickets, superworms, and soldier fly larvae. It is not yet understood what triggers EPN activation, or the contribution that activation has on IJ virulence in any given host. While we can suggest trends in virulence patterns, our data support the prior notion that the EPN infection process is multifaceted and no single bioassay will be predictive of virulence in all cases [8,9,10].

## 2. Materials and Methods

### 2.1. Nematode Production and Virulence Experiments

Infective juveniles of the species *Steinernema feltiae* (strain SN) and *Steinernema carpocapsae* (strain All) were propagated by infecting *Galleria mellonella* waxworms as previously described [15,16]. Fifteen waxworms were placed on top of a single sheet of 9 cm filter paper (Fisher 09-801B, Hampton, NH, USA) which was placed inside a 10 cm Petri dish. About 750 µL of nematodes (50 IJs per waxworm), and 150 µL of water was pipetted onto the filter paper with the waxworms. The infected waxworms were then incubated in the dark at 25 °C for a period of 7–10 days (7 for *S. feltiae*, and 10 for *S. carpocapsae*). Dead waxworms were then placed onto White traps in which a 5.5 cm filter paper (Fisher 09-795A) was placed on top of a 3.5 cm petri dish that was lightly wetted with tap water [15]. About 1 mL of tap water was pipetted to surround the 3.5 cm petri dish to ensure IJ emergence and survival. IJs emerged and moved into the water after 1–2 weeks of being on White traps. The IJs were collected and washed with tap water 3 times. This was done using a glass vacuum filter holder (Fisher Scientific, Cat# 09-753-1C) with two layers of 11 µm nylon filter nets (Millipore, NY1104700). The collection continued every day for a period of 14 days. IJs that were used for virulence were stored in tissue culture flasks at room temperature (~23 °C) and were used within 10 days. Virulence experiments were performed as described above for propagation, with some adjustments. We performed virulence assays on *Zophobas morio* (superworm), *Gryllus firmus* (sand cricket), and *Hermetia illucens* (soldier fly). Instead of using 10 cm petri dishes with multiple hosts, individual insects were placed into 6 cm Petri dishes. The total volume of liquid added to each dish was 400 µL, with different concentrations of IJs (100, 2000 and 5000 per 400 µL). Negative controls were given 400 µL of tap water. For superworm and soldier fly virulence assays, each experiment included 30 individual insects, where individual insects were placed in 6 cm petri dish arenas. These experiments were performed in triplicate, resulting in 90 individual insects being tested per concentration of nematodes. Each replicate was performed on a different day. For sand cricket assays, 15 individuals were used for each replicate, performed in triplicate, resulting in 45 individuals being tested per dose of nematode. Each replicate was performed on a different day.

### 2.2. Insects

Insects were purchased from Mulberry Farms, Fallbrook, CA, USA (http://www.mulberryfarms.com/), except for *G. firmus*, which was obtained from a breeding colony at the University of California, Riverside, originally collected from Florida in 1986 [17]. Insect storage and feeding different for each species. *G. mellonella* were kept in the dark at 18 °C until used. *Bombyx mori* and *Manduca sexta* were kept at 25 °C, and were fed commercial silkworm and hornworm chow, respectively, from Mulberry Farms. *Tenebrio molitor* and *Zophobas morio* were kept in the dark at 18 °C and fed carrots and apples ad libitum. *Gryllus firmus* and *Acheta domesticus* were kept at 25 °C and fed rabbit chow and water ad libitum. Insects used for virulence were stored for no longer than one month [13].

### 2.3. Making Homogenate from Various Insects

Homogenate was made as previously described [7,11], but without adding antibiotics. Briefly, frozen insects were ground up in liquid nitrogen using a mortar and pestle, yielding a fine powder, which was then transferred to a clean beaker. Phosphate-buffered saline was added to suspend the powder to reach the desired concentration (25% homogenate *w*/*v*). For example, 100 mL of 25% homogenate was made using 25 g of ground insect powder and 75 mL of PBS). The beaker was covered with plastic wrap and heated in a microwave oven until boiling. The mixture was then well-stirred and heated again to boiling. This was repeated 3–4 times in total. The homogenate was allowed to cool to room temperature. This mixture was then transferred to 15 mL or 50 mL conical tubes and centrifuged at 3000 rcf for 5–10 min at room temperature. The supernatant with the lipid layer (lipid content was higher for some insects than others) was collected in new tubes and centrifuged two more times to remove as much solid debris as possible. The final supernatant was mixed vigorously before being aliquoted and frozen at −20 °C for future use. Eight insect homogenates were produced: *G. mellonella* (waxworm), *B. mori* (silkworm), *M. sexta* (hornworm), *G. firmus* (sand cricket), *A. domesticus* (house cricket), *T. molitor* (mealworm), *Z. morio* (superworm), and *H. illucens* (soldier fly). For each of these the homogenate was made diluted with PBS to be 25% crude.

### 2.4. Activation of IJs and Quantification

The method used to activate IJs was done as previously described [7,11], with some modifications. Sponge foam (Drosophila plugs, Biologix 51–17725) was cut into ~3 mm × 3 mm × 10 mm pieces. Then 0.08 g of sponge pieces were soaked in 1 mL of insect homogenate. The homogenate we used was not supplemented with antibiotics. A homogenate to sponge ratio of 12:1 (volume/weight) was used to obtain a balance between food content and aeration in the sponge matrix. The soaked sponge was then piled into a 35 mm petri dish and approximately 5000 IJs were pipetted onto the homogenate-soaked sponge. The uncovered petri dish was incubated in the dark at 25 °C for 6 h. Activation occurs in a temporal manner [7,11]. Previous research has demonstrated that the proteins released by *S. carpocapsae* IJs activated for 6 h are highly toxic [7], and that more than 75% of *S. carpocapsae* IJs are releasing their symbiotic bacteria after 6 h of exposure to hemolymph [18]. Based on these previous studies we decided to focus on the 6 h time point. The IJs were counted and quantified as previously described [7,11]. The IJs used in the activation experiment were 2–14 days old (post-emergence). For each replicate, 150–300 nematodes of the 5000 used were assessed for activation. Each experiment was done in triplicate and on different days, resulting in 450–900 individual *S. carpocapsae* and *S. feltiae* nematodes being assessed per insect homogenate used. To assess the effects of age on IJ activation, nematodes were collected in the following way: Two days after White traps were set up, all the IJs that had emerged were removed along with all of the water in the trap and both the water and IJs were discarded. Fresh water was then added and IJs were collected every two days thereafter until 14 days after the initial setup of the White trap. The trap was completely cleaned upon each collection, with all of the IJs and water being removed, and fresh water being added. IJ age was counted as the number of days elapsed post-emergence. Young IJs were less than 10 days post-emergence while old IJs were greater than two weeks post-emergence.

### 2.5. Statistical Analysis

Activation and survival assays were graphed and analyzed using Graphpad Prism software (GraphPad Software, Inc., La Jolla, CA, USA). The *p*-values were obtained from the mantel-cox log-rank test for the virulence assays, 2-way ANOVA test for the activation assays, and a paired *t* test for the age-evaluated activation assays. We used Sidak’s multiple comparisons test to compare *S. carpocapsae* and *S. feltiae* activation for each insect homogenate. The statistical values for all assays are reported in the results section.

## 3. Results

### 3.1. IJ Activation is Species Specific and Host Specific

**Figure 1:**
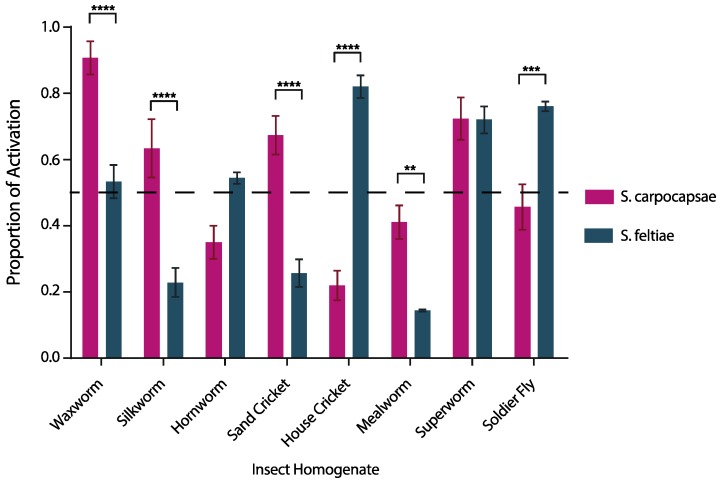
Activation of *S. carpocapsae* and *S. feltiae* infective juveniles (IJs) when exposed to different host homogenates. Infective juveniles were exposed to each homogenate before being quantified. Values represent the proportion of IJs that were activated after 6 hours of exposure to host homogenate and data were analyzed using the 2-way ANOVA test in GraphPad Prism software (Table A1). Comparisons between *S. carpocapsae* and *S. feltiae* for different homogenates were assessed using Sidak’s multiple comparisons test. The means from three independent experiments are shown and bars represent standard errors. ** *p* < 0.01, *** *p* < 0.001, **** *p* < 0.0001.

We exposed *S. carpocapsae* and *S. feltiae* IJs to eight different insect host homogenates. We found that IJ activation is species-specific and host-specific (Figure 1). *Steinernema feltiae* demonstrated highest activation when exposed to house cricket homogenate and lowest activation when exposed to mealworm homogenate (Figure 1). Whereas *S. carpocapsae* demonstrated highest activation when exposed to waxworm homogenate and lowest activation when exposed to house cricket homogenate (Figure 1). We found that homogenates from waxworms (*p* < 0.0001), silkworms (*p* < 0.0001), house crickets (*p* < 0.0001), soldier flies (*p* < 0.001), mealworms (*p* < 0.01), and sand crickets (*p* < 0.0001) yielded significant differences in the proportion of IJs activated between *S. carpocapsae* and *S. feltiae*. *Steinernema carpocapsae* and *S. feltiae* had similar levels of activation when exposed to homogenates from hornworm (*p* = 0.0986) and superworm (*p* = 0.999). The results of the two-way ANOVA are reported in Table A1.

### 3.2. IJ Activation Is Affected by IJ Age

**Figure 2:**
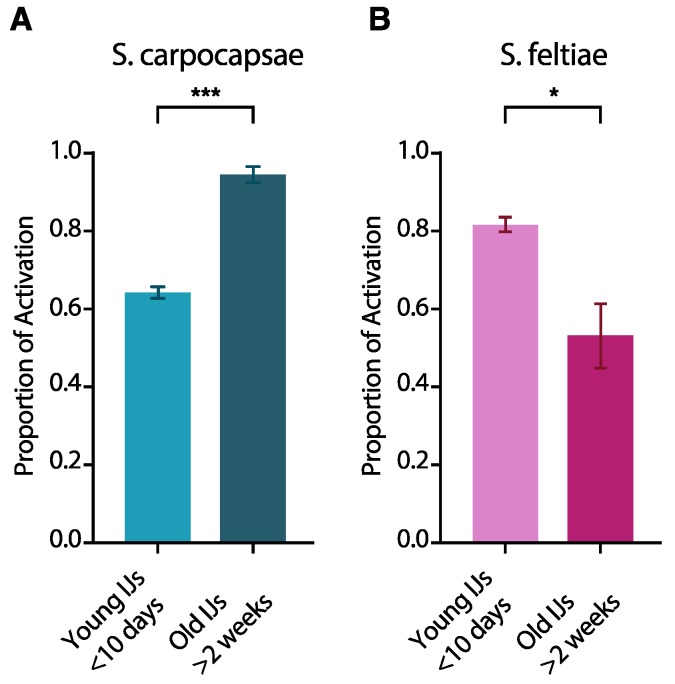
The effect of IJ age on activation. (**A**) *S. carpocapsae* IJs; (**B**) *S. feltiae* IJs. Populations of IJs were split into two groups based on age: IJs that had emerged less than 10 days prior to experimentation and IJs that had emerged at least 2 weeks prior to experimentation. Data was analyzed using the paired *t*-test in GraphPad Prism software. The means from three independent experiments are shown and bars represent standard errors. * *p* < 0.05, *** *p* < 0.001.

Next, we evaluated the effects of age on IJ activation for both *S. carpocapsae* and *S. feltiae* IJs when exposed to waxworm homogenate for 6 hours, by testing IJs that were less than 10 days old (post-emergence) and compared them to IJs that were more than 2 weeks old. We found that IJ age significantly affects activation in both EPNs. A higher proportion of older *S. carpocapsae* IJs activated than young *S. carpocapsae* IJs. In contrast, a significantly higher proportion of young *S. feltiae* IJs activated than older *S. feltiae* IJs (Figure 2).

### 3.3. In Vitro IJ Activation Alone Is not Always Predictive of Virulence

**Figure 3:**
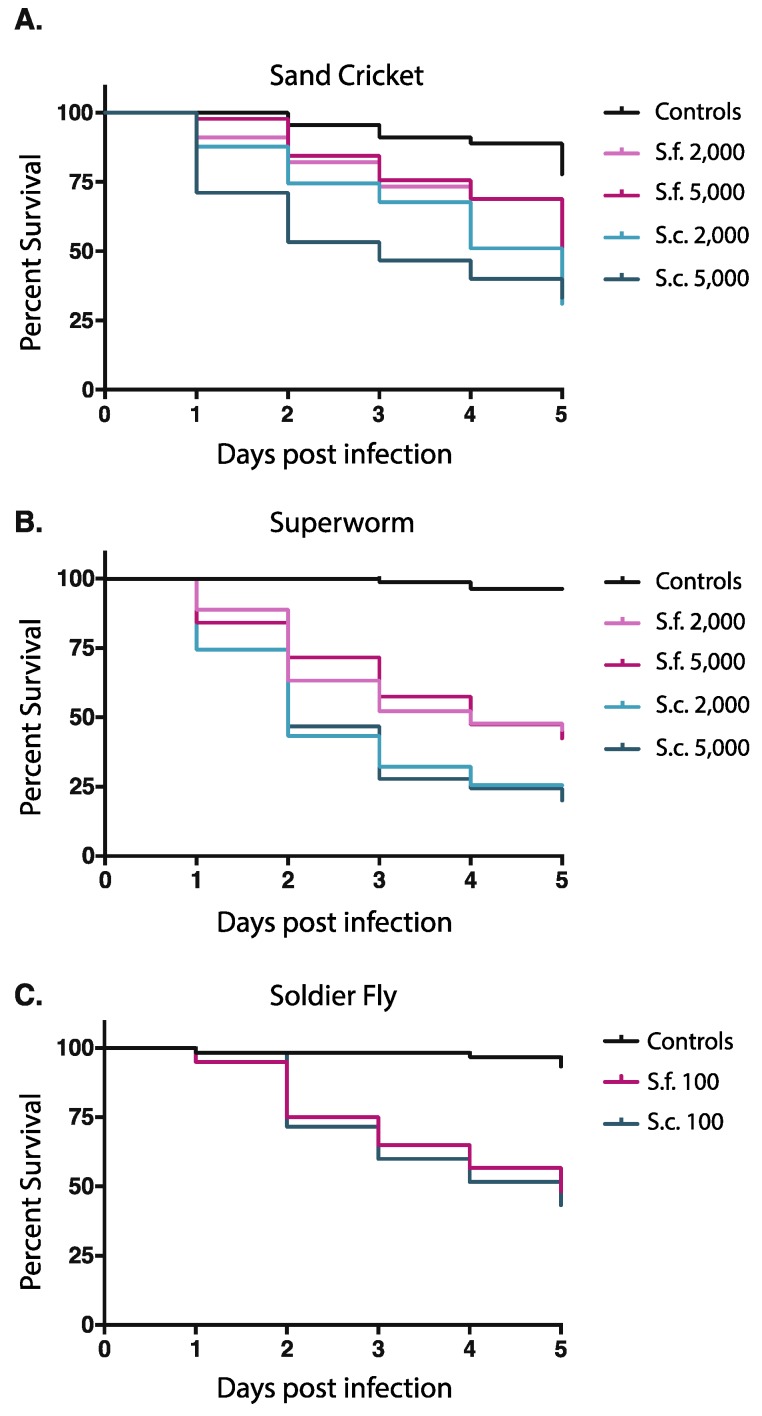
Survival of host insects after exposure to different doses of *S. carpocapsae* or *S. feltiae* IJs. (**A**) Sand cricket adults exposed to nematode IJs; (**B**) Superworm larvae exposed to nematode IJs; (**C**) Soldier fly larvae exposed to nematode IJs. Hosts exposed to water but no IJs served as controls. Survival was monitored every 24 h up to 120 h post-exposure. Values represent the total of three experiments performed for each host and dose. Data was analyzed using log-rank analysis in GraphPad Prism software.

To better understand the role of activation in EPN infections, we evaluated the virulence of *S. carpocapsae* and *S. feltiae* against three insect hosts: Sand crickets, superworms, and soldier fly larvae. Mortality was quantified for a period of 5 days post-infection. First, we examined the virulence of these EPNs against adult sand crickets. Because we observed higher activation of *S. carpocapsae* IJs in sand cricket homogenate, we hypothesized that it would be more virulent to sand crickets than *S. feltiae*. Both EPN species we tested were capable of infecting and killing sand crickets, although it required relatively high doses of nematodes (≥2000 IJs). There was a significant difference between the least virulent exposure (2000 *S. feltiae*) and controls (*p* = 0.0102). As predicted by the activation data, *S. carpocapsae* was significantly more virulent to sand crickets than *S. feltiae* at both doses that we tested (Figure 3A; *p* = 0.0216 and 0.0261, respectively).

Notably, exposing sand cricket adults to 5000 *S. feltiae* IJs was not significantly more lethal than exposure to 2000 IJs (Figure 3A; *p* = 0.6898). This observation was also consistent for *S. carpocapsae* infections; exposing sand crickets to 5000 *S. carpocapsae* IJs was not significantly more virulent than exposure to 2000 IJs (Figure 3A; *p* = 0.3880).

Next, we tested IJ virulence in superworms. Since we observed equal activation for *S. carpocapsae* and *S. feltiae* IJs in superworm homogenate, we predicted there would be no significant difference in virulence. Both EPN species we tested were capable of infecting and killing superworms (Figure 3B), but relatively high doses of IJs were required (≥2000 IJs), as was observed for sand cricket infections. Contrary to our prediction, *S. carpocapsae* was significantly more virulent than *S. feltiae* at both the 2000 and 5000 dosages (Figure 3B; *p* = 0.0016 and 0.0003, respectively). As was observed in sand crickets, exposing 5000 *S. carpocapsae* IJs to superworms did not lead to significantly higher mortality than exposure to 2000 IJs of the same species. Exposing 5000 *S. feltiae* IJs to superworms also did not lead to significantly higher mortality than exposure to 2000 IJs of the same species. (Figure 3A,B).

We then tested IJ virulence in soldier fly larvae. Because *S. feltiae* IJs activated better in soldier fly homogenate, we predicted that *S. feltiae* would be more virulent than *S. carpocapsae* against this host. We found both EPN species were virulent at much lower doses (100 IJs) than were required to kill sand crickets and superworms. Negative controls persisted significantly better than the least virulent dose (Figure 3C; *p* < 0.0001). Contrary to our prediction based on activation, there was no significant difference in virulence between *S. feltiae* and *S. carpocapsae* IJs (Figure 3C; *p* = 0.6244).

## 4. Discussion

### 4.1. The Activation Bioassay Illustrated Species-Dependent Nematode Activation among a Range of Hosts

Infection by EPNs is the result of a complex process that includes long- and short-range volatile chemical and contact cues, penetration into the host, activation (also known as IJ recovery), release of the symbiotic bacteria, and the release of excreted/secreted nematode proteins (ESPs), which culminates in the death of the host [9,10,19]. Activation is a key aspect of the EPN infection process and is required for efficient release of the pathogenic bacteria they carry along with any nematode-derived bioactive molecules that function in killing the host or modulating its immune response [7,20,21]. Despite the importance of activation, it has not been well studied among EPNs, nor has it been previously considered as a means of predicting EPN virulence. A quantitative activation bioassay has been used recently to reaffirm that *S. scapterisci* is indeed a cricket specialist [11]. This protocol was designed to mimic the environment a nematode would experience within a host and has previously been used to study the identity and role of nematode excreted/secreted proteins in EPN infection [7,22,23,24]. We used this bioassay to determine whether the activation process was similar for different EPNs and to determine its usefulness in predicting EPN virulence. Our data suggest that EPN IJ activation is a species-specific and host-specific process and that nematode host preferences coincide, at least partially, with activation levels in that host. We observed that *S. carpocapsae* and *S. feltiae* are differentially activated in some hosts while similarly activated in others. Having identified these observed differences in activation, we wanted to determine the predictive value of in vitro activation on EPN virulence.

### 4.2. Age Affects IJ Activation

It is well known that age has a significant effect on many IJ traits considered important for infection including host-seeking behavior and penetration [25,26]. Particularly relevant to the data we have presented is the observation of phased infectivity in EPN IJs, the phenomenon described in both heterorhabditids and steinernematids where the tendency of IJs to infect changes as they age (time from IJ emergence increases) [10,27,28]. Here, we reported that age affects activation in both *S. carpocapsae* and *S. feltiae*, but in different ways. We found that the proportion of *S. carpocapsae* IJs that activate when exposed to waxworm homogenate was significantly higher in older IJs, whereas we observed the opposite in *S. feltiae*. The implications of these findings are difficult to interpret at this time, due at least in part to the nature of the experiments themselves and the position of activation in the process of infection. Unlike previous studies that have measured the effects of IJ age on attraction to hosts or their decision to infect hosts [10,25,26], we measured activation after exposure to insect homogenate; essentially circumventing the nematodes’ ability to choose to move toward or even to penetrate hosts. There are earlier steps in the process of infection during which the nematodes may decide not to continue to infect and therefore avoid the decision of whether or not to activate. We speculate that differences in host tissue/homogenate provide important information to EPN IJs that influence activation in a context-specific manner. The decision to invade a host is irreversible, because development is resumed, and morphological changes begin to take place [7,11,29]. We note that the experimental conditions we used may have favored activation, since the nematodes were exposed to host tissue without long-range or short-range cues in these experiments, while in nature they may simply choose not to move toward or enter certain hosts. Nevertheless, we found that IJ activation upon exposure to host tissue is affected by IJ age and this may be useful to future studies on activation.

### 4.3. In Vitro Activation alone May Be Predictive of Virulence in Some Cases

EPN activation is an important part of the process of infection and a deeper understanding of the cues used by different EPN species to inform this process may be useful. We determined that for six of the eight insects we used, there is a significant difference in the proportion of *S. carpocapsae* and *S. feltiae* IJs that activate. We hypothesized that IJ activation may be used to predict virulence and we tested this hypothesis in three different insects: (1) Sand crickets, where a significantly higher proportion of *S. carpocapsae* IJs activated; (2) superworms, where there was no significant difference between *S. carpocapsae* and *S. feltiae* activation; and (3) soldier flies, where a significantly higher proportion of *S. feltiae* IJs activated. We found that activation alone is not always predictive of virulence, but for one of the three hosts we tested, the sand cricket, higher activation did correlate with higher virulence. However, there are many factors that contribute to EPN IJ virulence including nematode behavior, host behavior, host immunity, nematode penetration into the host, activation, and the contribution of the bacterial symbiont, among others. Below we consider how some of these factors may explain why in vitro activation alone is not always predictive of activation.

Bacterial symbionts and nematode-derived molecules each influence EPN virulence and make species-specific contributions. EPNs release their bacterial symbionts and secrete their toxins only after being activated inside the host. EPN virulence is dependent on its own toxic secretions and/or bacterial symbiont, depending on the species. Certain EPN symbionts have shown attenuated virulence when injected into different insect hosts [20,21,30], and the total number of bacterial cells carried by each individual IJ varies from species to species, where, for example, *S. carpocapsae* IJs carry approximately 40 colony forming units (CFUs) each while *S. scapterisci* IJs carry approximately 8 each or fewer [21,31]. Therefore, a well-activated nematode is not necessarily lethal to its host, rather the overall virulence depends on the quantity and toxicity of the bacterial symbiont as well as the potency of host immunity, and the toxicity of the nematode-derived excreted/secreted proteins, among other factors.

Penetration is another factor that may affect virulence [32,33]. Specifically, *S. carpocapsae* and *S. feltiae* entry into the host, a precursor to activation, has been shown to be not only different in the same host but also correlated to insect mortality in select examples [32]. While species-specific penetration rates have been described, it has also been found that penetration rates can significantly vary within populations of the same species [34]. We found that *S. carpocapsae* and *S. feltiae* were similarly activated when exposed to superworm homogenate. Yet, *S. carpocapsae* was significantly more virulent towards superworms than *S. feltiae*. In soldier fly larvae, there were significantly fewer *S. carpocapsae* IJs that activated than we observed for *S. feltiae*, yet the overall virulence was similar for both species. We note that superworms and sand crickets required unusually high numbers of IJs to be killed at an appreciable rate (>2000 IJs), suggesting either that these species are poor hosts for *S. carpocapsae* and *S. feltiae*, or that they are more resistant to infection by these nematodes than most insects that have been tested. Further research is needed to determine which steps of the infection process are difficult to achieve when *S. carpocapsae* and *S. feltiae* attempt to infect superworms and sand crickets. Nematode activation is only one part of a multi-step host-killing process. It is possible that penetration rates are a factor that explains why activation is only predictive of virulence in some cases, but this is difficult to determine given the variability of penetration rates between and within species.

One interesting possibility is that activation is predictive of virulence in cases where some minimum threshold of activation is not met. For example, we observed that approximately 70% of *S. carpocapsae* IJs activated within 6 hours of exposure to sand cricket homogenate while less than 30% of *S. feltiae* IJs activated in that same time period. In this case *S. carpocapsae* IJs were significantly more virulent than *S. feltiae* IJs. However, in both the superworm and soldier fly, at least 50% of the IJ population of both *S. carpocapsae* and *S. feltiae* activated, meaning that at least half of the IJs were releasing bacterial pathogens and excreting/secreting proteins. Perhaps once some threshold of activation is met, other factors become more important to virulence such as bacterial pathogenesis or the potency and/or specificity of nematode-derived toxins and immune modulators. More experimentation is necessary to fully test this hypothesis.

## 5. Conclusions

The quest to find, kill, and reproduce in a host is long for EPNs. EPNs must sense the host, penetrate the host, survive and activate in the host, kill the host, and then finally reproduce in the host [9]. Our data suggest that activation alone is not always predictive of virulence. However, low activation rates may be predictive of decreased virulence as we observed with *S. feltiae* in the sand cricket. While high levels of activation are not predictive of virulence, a deficiency in activation or any other part of the infection process may lead to diminished virulence.

It has been noted that one major challenge faced by researchers studying EPNs is to determine which behaviors might be the most important to document [19]. Certainly, this depends on what is being studied and whether the focus is aimed towards developing EPNs in biological control or improving our general understanding of EPN biology. Here we used in vitro nematode activation as a metric to predict virulence. Our data suggest that activation may be useful in predicting virulence, especially when coupled with one or more additional factors such as nematode attraction or penetration. Our understanding of the molecular signals involved in activation and how it relates to virulence, development, and reproduction remains limited. Additional research is necessary to clarify these details and relate what is known in the laboratory to EPN interactions in soil.

## Appendix A

**Table A1:** Summary statistics resulting from the two-way ANOVA performed on the activation data presented in Figure 1. This analysis was performed in Graphpad Prism software.

**Source of Variation**	**% of Total Variation**	***p*** **Value**	**Summary**		
Interaction	55.57	<0.0001	****		
Row Factor	35.84	<0.001	****		
Column Factor	0.9130	0.0671	ns		
**ANOVA table**	**SS**	**DF**	**MS**	**F (DFn, DFd)**	***p*** **value**
Interaction	1.515	7	0.2164	F (7, 31) = 31.31	*p* < 0.001
Row Factor	0.9772	7	0.1396	F (7, 31) = 20.20	*p* < 0.001
Column Factor	0.02489	1	0.02489	F (1, 31) = 3.601	*p* = 0.0671
Residual	0.2143	31	0.006912		

*** *p* < 0.001, **** *p* < 0.0001, ns = not significant. SS = sum-of-squares, DF = degrees of freedom, MS = mean squares, F = F ratio, DFn = degrees of freedom of the numerator, DFd = degrees of freedom of the denominator.
